# Impact of Video Laryngoscopy on Advanced Airway Management by Critical Care Transport Paramedics and Nurses Using the CMAC Pocket Monitor

**DOI:** 10.1155/2015/821302

**Published:** 2015-06-17

**Authors:** Bradley Boehringer, Michael Choate, Shelley Hurwitz, Peter V. R. Tilney, Thomas Judge

**Affiliations:** ^1^LifeFlight of Maine, 13 Main Street, Camden, ME 04843, USA; ^2^Laurea University of Applied Sciences, Uudenmaankatu 22, 05800 Hyvinkää, Finland; ^3^Brigham and Women's Biostatistics Center, 5 Francis Street, Boston, MA 02115, USA

## Abstract

Accurate endotracheal intubation for patients in extremis or at risk of physiologic decompensation is the gold standard for emergency medicine. Field intubation is a complex process and time to intubation, number of attempts, and hypoxia have all been shown to correlate with increases in morbidity and mortality. Expanding laryngoscope technology which incorporates active video, in addition to direct laryngoscopy, offers providers improved and varied tools to employ in management of the advanced airway. Over a nine-year period a helicopter emergency medical services team, comprised of a flight paramedic and flight nurse, intended to intubate 790 patients. Comparative data analysis was performed and demonstrated that the introduction of the CMAC video laryngoscope improved nearly every measure of success in airway management. Overall intubation success increased from 94.9% to 99.0%, first pass success rates increased from 75.4% to 94.9%, combined first and second pass success rates increased from 89.2% to 97.4%, and mean number of intubation attempts decreased from 1.33 to 1.08.

## 1. Introduction

Prehospital advanced airway management by paramedics and nurses has become an increasingly relevant and debated topic. Research has persistently demonstrated that failure rates of prehospital transport personnel are far higher and fraught with more complications compared to those of in-hospital personnel or physician based helicopter EMS (HEMS) colleagues [1, 2]. In cases such as cardiac arrest, recently published data is beginning to show that management with supraglottic airways or a bag-valve mask may be effective, especially in cases where immediate airway protection by endotracheal tube (ETT) is unlikely or apt to be accompanied by adverse events [3–6].

The North American HEMS crew configuration of a nurse and paramedic is atypical when compared with the international air medical industry. Research shows that critical care flight crews in this configuration manage the airway more successfully than their ground counterparts [7] and often quite similar to that of their physician colleagues who document ETT successes of between 95% and 99.2% [8, 9]. While there is a clear correlation between successful airway management and volume of exposure, the impact of aggressive education and QI processes remain unclear [10]. Furthermore rapid sequence induction protocols appear to improve first pass success of prehospital providers [10–16], as does video laryngoscopy, especially with respect to difficult airways [17, 18]. Video laryngoscopy has demonstrated shorter entry to POGO (percentage of glottic opening) and entry to tube times, improved glottic view, and lower incidence of esophageal ETT placement [19–22].

The gold standard for successful airway management continues to be the ability to insert an ETT on the first attempt with minimal or no adverse sequela such as hypoxia or hypotension. It has been shown that adverse events and failure rates increase with repeat attempts at intubation [23–25] and occur more quickly between the first and second attempts. In fact, a single repeat intubation attempt increases the risk of experiencing an adverse event from 14% to 47% [23].

The intent of this retrospective chart review and analysis was to determine the impact of adding video laryngoscopy on markers related to effective prehospital airway management by a North American critical care transport team. Primary clinical indicators were first pass success, combined first and second pass success, ultimate method of securing the airway, and need for rescue airways such as supraglottic devices and cricothyrotomy.

## 2. Methods

This was a retrospective chart review of intubations performed by critical care flight paramedics and nurses from 2006 through the third quarter of 2014. Intubation attempts were defined as laryngoscopy with intent to place an advanced airway. For example, if laryngoscopy was aborted and a device would have been placed had a view been possible, this was considered an attempt.

Descriptive data was evaluated at 99% confidence intervals (except where noted) and chi square testing using Fisher's exact test was completed for significance. Raw data is shown in the tables.

Due to the retrospective, quality improvement nature of the data collection the local IRB Committee deemed that this paper did not require approval.

## 3. Setting

Advanced airway management is done by flight practitioners in a nurse/medic configuration employed at a moderately sized critical care transport organization in the northeast of the United States. The company currently operates two Agusta 109E rotor wing aircraft that offer primary coverage for over 33,000 square miles and 274 first response agencies and interfacility transport services for 38, mostly rural, hospitals. Call volume averages have steadily risen and currently top 1600 each year with approximately 23% scene and 77% interfacility missions though advanced airway encounters are seen more often in scene responses, 58% versus 42%, respectively.

The flight team staff includes both paramedics and nurses who are chosen based upon prior relevant critical care experience. Once selected, all staff receives the same crew member orientation and advanced airway preparation. Prior to autonomous performance on missions crew members must complete a comprehensive advanced airway management education. It minimally includes ten operating room and in situ intubations, an advanced airway skills lab, and quarterly, service-wide QI meetings. Upon completion of orientation, crews are mandated to complete at least two live intubations on adults and one pediatric intubation, which may be performed on a manikin, each quarter. Yearly education requires revisiting the airway lab for updates on advanced airway management which includes surgical airway review and other advanced ventilatory skills. All crew members receive peer to peer and medical director chart review after each flight. Feedback includes medication management of the advanced airway during and after intubation, troubleshooting techniques, and overall success and performance. Rapid sequence intubation (RSI) has been protocolized for the flight practitioners and includes the most current practices in medication administration, adjunct and rescue devices, and general airway management techniques which all may be used during advanced airway management at the discretion of the crew. During the study window there were no significant changes in crew configuration or training.

Until early 2013, direct laryngoscopy was the routine approach used to visualize the vocal cords when securing an advanced airway. Rarely did crews encounter a video laryngoscope of any type at sending facilities. In 2013 the program placed the Karl Storz CMAC Pocket Monitor Video Laryngoscope into service as the primary visualization device. The CMAC was chosen because, unlike many other video laryngoscopes, its shape most closely mimics a traditional Macintosh blade allowing for either a direct or video view, allowing crews to maintain a technique similar to that of traditional laryngoscopy. Standard Macintosh blades sizes 2 and 4 were placed on each aircraft. Intermediate sizes were not chosen due to cost constraints and as such a full set of traditional laryngoscopy devices continue to be carried.

## 4. Selection of Participants

All patients requiring intubation by flight crews from 2006 through the third quarter of 2014 were included. No distinction was made between ground and flight missions. If the patient expired during a flight crew interaction, and an attempt was made to secure the airway, the experience was included in the study. Both RSI and non-RSI cases were included in the data analysis.

## 5. Data Collection

Data collection, as part of a robust quality improvement process, has been through thorough review of electronic patient care records. Each patient care record was reviewed for quality markers and patient deterioration. All encounters requiring airway and ventilatory support greater than free flow oxygen were separately screened for decision to intubate based on physiologic markers. Excel spreadsheets were used for primary analysis and to organize data.

## 6. Results

Total mission volume (ground and air transport) during the study period was 12,361 with 790 advanced airway encounters (6.4%). Two airway encounters were not included in the data review as the GlideScope was used. Initial data analysis showed a gender breakdown of 69% male and 31% female encounters, 60% trauma and 40% medical patients, and 94.3% adult (>13 years of age), 2.8% pediatric (< or = 13, >1 year of age), and 1.4% infant patients (<1 year of age). 1.5% had no age recorded (*n* = 12). See Table 1.

### 6.1. Successful Endotracheal Intubations by Flight Crew

**Figure 1:**
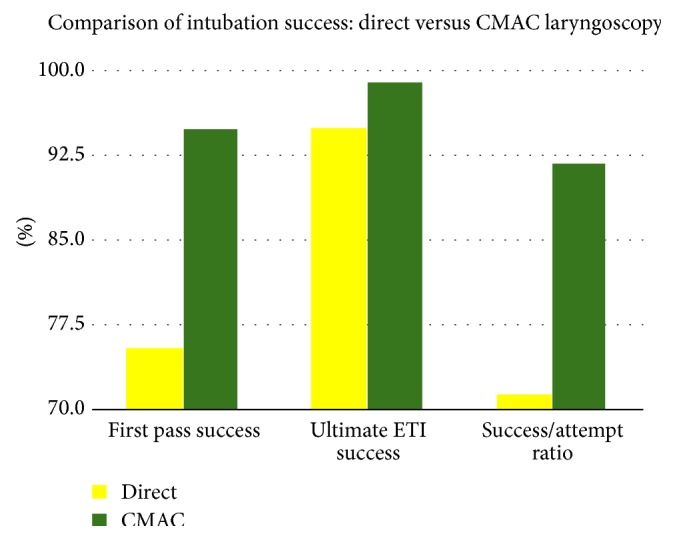
Comparison of intubation success using data from Table 2.

After the implementation of the CMAC, overall endotracheal intubation success by a critical care transport practitioner increased from 94.9% to 99.0% (significant at CI 95%, chi square = 6.13, Fisher's exact test *P* = 0.011), first pass success rates increased from 75.4% to 94.9% (significant at CI 99%, chi square = 35.12, Fisher's exact test *P* < 0.0001), combined first and second pass success rates increased from 89.2% to 97.4% (significant at CI 99%, chi square = 12.44, Fisher's exact test *P* = 0.0002), and the success to total attempts ratio increased from 71.4% to 91.9% (significant at CI 99%, chi square = 38.05, Fisher's exact test *P* < 0.0001). See Figure 1. While overall and first attempt success adequately reflect a system's exemplary performance, the success to attempt ratio specifically illuminates what is happening in other cases where there was no success, or more than one attempt was required. An alternative view of the success to total attempts ratio would be that, respectively, one extra attempt was required in 3.5 patients, with improvement to one extra attempt in 11 patients. Mean number of intubation attempts for all airway encounters, successful or unsuccessful, decreased from 1.33 (*n* = 593) with direct laryngoscopy to 1.08 (*n* = 195) when using the CMAC. The reduction was statistically significant using a two-tailed *t*-test (CI 95%, *t* = 6.21, DF 578.12, *P* < 0.0001). There were no attempts to use video laryngoscopy after failed direct laryngoscopy.

### 6.2. Unsuccessful Intubations by Flight Crew and Outlying Events

In flight intubation occurred in only two instances and both were successful (0.25%).

For patients unable to be intubated with an ETT, supraglottic devices (LMA or King Airways) were placed in 19 cases after direct laryngoscopy (3.2%) and in 1 instance after video laryngoscopy with the CMAC (0.5%). The reduction in supraglottic device use is significant (CI 95%, chi square = 4.297, Fisher's exact test *P* = 0.036).

Other providers (CRNA, MD, and on-scene paramedic) secured the airway after initial attempted management by flight crews in 7 cases status after direct laryngoscopy (1.2%) and in 0 cases after video laryngoscopy (0%) which was not significant (CI 95%, chi square = 2.323, Fisher's exact test = 0.2028). Cricothyrotomy was required in two cases of failed direct laryngoscopy (0.3%, both were performed by flight crew) and in one case after failed video laryngoscopy (0.5%, performed by hospital surgeon). The use of cricothyrotomy was not statistically significant (CI 95%, chi square = 0.119, Fisher's exact test *P* = 0.574). See Table 2 for complete results.

## 7. Discussion

Air medical providers are consistently called for the most critically unstable patients in prehospital and rural primary hospital care. As such, their training must reflect an attempted mastery of the requisite skills, but more importantly, they must maintain a procedural proficiency necessary to care for patients in an extremely dynamic environment. Airway management is one of these required skills. In recent years prehospital personnel, who have historically seen advanced airway management as part of their standard skill set, have come under increased scrutiny due to worse outcomes when compared with physician counterparts [26]. A 2014 Dutch report lists nonflight trained paramedic first pass success rates at just over 46% [1]. Other reports are equally concerning. Time spent securing the airway, often while neglecting other important tasks, failed attempts, and adverse outcomes have each caused programs, regions, and countries to reevaluate policies around advanced airway management. Often this has left agencies with no other choice than to adopt basic life support level airway management skills, often in the form of blind insertion devices. Skills proficiency and retraining are typically easier and more quickly achieved with these devices. This seems especially prudent in settings where advanced airway management is a rarely practiced skill. Understanding these dynamics is crucial in picking the most appropriate approach to airway management.

Despite an unchanging approach to the process of airway management over the nine-year study period, this agency demonstrated dramatic increases in successful airway management after the implementation of the CMAC video laryngoscope. Improvements were seen in all primary measures of advanced airway success: ultimate endotracheal intubation success, first pass success, combined first and second pass success, success to total attempt ratios, mean attempts, and incidence of supraglottic device use. Historically this critical care transport team demonstrated ultimate endotracheal intubation and first pass success at 94.9% and 76.6%, respectively, which is similar to other internationally reported figures for flight practitioners in the US [11]. Current success and first pass success rates with the CMAC have improved to 99% and 94.9%, respectively. When compared to our historic data prior to the use of the CMAC previous studies have shown higher mean attempts with direct laryngoscopy. The use of video laryngoscopy, however, seems to decrease mean intubation attempts in all patient encounters [27].

In comparison, European critical care teams, whether ground or air based, are more commonly led by a physician and in many cases a physician is required to be present during an intubation attempt. Ultimate intubation success by European physicians is most commonly reported to be between 96 and 99% [1, 8, 28, 29] but as low as 88% [30]. Physician first pass success tends to hover near 85% [1, 31] with a low percentage of 68% [29]. In most cases these reports are at minimum equal to, or worse than, the findings in our data review. This retrospective chart review demonstrates that a US based air ambulance staffed with critical care nurses and paramedics is able to achieve similar, if not better, rates of first pass and overall intubation success with the assistance of video laryngoscopy, in this case the CMAC.

## 8. Limitations

While our data suggests that the CMAC may have an impressive impact on intubation success, the review certainly has limitations. Some of these are inherent to retrospective reviews and others specific to the human bias in documentation and data collection. Without in situ video documentation of an airway encounter, one can never be certain how many attempts were actually needed to secure an airway, how long it took, or what view was actually obtained. Furthermore defining what counts as an attempt can be equally challenging as some providers may only count attempts at actually placing an endotracheal tube and not the “first look.” Strict defining guidelines typically include any instance when a provider places the laryngoscope blade into a patient's mouth but, again, this is hard to ascertain without an independent observer being present or video recording of an encounter that can be independently reviewed at a later date. Our currently reviewed data also fails to include adverse events such as hypotension and hypoxia as these points were not part of historic data collection or the QI process. Other potentially confounding variables that were not taken into account were the induction medications, or lack thereof, and the use of adjuncts such as an introducer.

## 9. Conclusions

Expanding video laryngoscope technology is offering providers new tools to employ in the management of advanced airways. Options have become more compact and less cost prohibitive for prehospital programs. The body of data to support their use is growing and is supported by this program's experience with the CMAC. While certainly only a piece of the complex puzzle in advanced airway management, clinical markers were significantly improved after its implementation. A robust training program, both initial and ongoing, a routine QI process, and RSI protocols are likely crucial contributors to success in advanced airway management.

## Figures and Tables

**Table 1:** General summary of airway encounter data.

Age	
Adult (>13)	743 (94.30)
Pediatric (<14, >1)	22 (2.8%)
Infant (<1)	11 (1.4%)
Unknown	12 (1.5%)

Sex	
Male	544 (69.4%)
Female	241 (30.5%)
Not documented	3 (0.38%)

Interfacility	331 (42.0%)
Scene	457 (57.97%)

Trauma	473 (60%)
Medical	315 (40%)

^∗^Two GlideScope encounters are not included.

**Table 2:** Summary of advanced airway encounters.

	Direct laryngoscopy	CMAC
Total encounters (*n* = 790) ^∗^2 GlideScope encounters not included	593	195
Total attempts	789	210
Ultimate ETT success	563	193
Mean attempts	1.33	1.08
First pass success	447	185
First and second pass	529	190
Supraglottic device use	19	1
Other providers secured	7	0
Patient pronounced after 1 attempt (failed ETI)	2	0
Cricothyrotomy	2	1
